# Age‐related differences in agility are related to both muscle strength and corticospinal tract function

**DOI:** 10.14814/phy2.70223

**Published:** 2025-02-21

**Authors:** Evan G. MacKenzie, Nick W. Bray, Syed Z. Raza, Caitlin J. Newell, Hannah M. Murphy, Michelle Ploughman

**Affiliations:** ^1^ Recovery and Performance Laboratory (Division of Biomedical Sciences, Faculty of Medicine Memorial University of Newfoundland) St. John's Newfoundland and Labrador Canada

**Keywords:** age, agility, corticospinal tract, neuromuscular system, transcranial magnetic stimulation

## Abstract

Agility is essential for “healthy” aging, but neuromuscular contributions to age‐related differences in agility are not entirely understood. We recruited healthy (*n* = 32) non‐athletes (30–84 years) to determine: (1) if aging is associated with agility and (2) whether muscle strength or corticospinal tract function predicts agility. We assessed muscle strength via a validated knee extension test, corticospinal tract function via transcranial magnetic stimulation, and agility via spatiotemporal values (i.e., leg length‐adjusted hop length and hop length variability) collected during a novel propulsive bipedal hopping (agility) task on an electronic walkway. Pearson correlation revealed aging is associated with leg length‐adjusted hop length (*r* = −0.671, *p* < 0.001) and hop length variability (*r* = 0.423, *p* = 0.016). Further, leg length‐adjusted hop length and hop length variability correlated with quadriceps strength (*r* = 0.581, *p* < 0.001; *r* = −0.364, *p* = 0.048) and corticospinal tract function (*r* = −0.384, *p* = 0.039; *r* = 0.478, *p* = 0.007). However, hierarchical regressions indicated that, when controlling for sex, muscle strength only predicts leg length‐adjusted hop length (*R* ^2^ = 0.345, *p* = 0.002), whereas corticospinal tract function only predicts hop length variability (*R* ^*2*^ = 0.239, *p* = 0.014). Therefore, weaker quadriceps decrease the distance hopped, and deteriorating corticospinal tract function increases variability in hop length.

## 1 INTRODUCTION

Agility helps us navigate complex and unpredictable environments; it reflects an ability to quickly change one's body position, relying on strength, power, endurance, balance and coordination, and reaction time (Morat et al., 2020). Even “normal” or typical aging, which can begin as early as the fourth decade, triggers complex changes in the neuromuscular system, potentially impairing agility (Hunter et al., 2016; McNeil & Rice, 2018; Wu et al., 2016). Age‐related neuromuscular decline is associated with worse health outcomes (Buch et al., 2016; Cadore et al., 2019; Swiecicka et al., 2019). However, such decline may be delayed given that exercise programs directly or indirectly incorporating agility training improve balance (Lichtenstein et al., 2020; Liu‐Ambrose et al., 2004; Tollar et al., 2019), reduce fall risk (Donath et al., 2016; Reed‐Jones et al., 2012; Rodrigues et al., 2023), enhance quality of life (Galan‐Arroyo et al., 2022; Liu‐Ambrose et al., 2005; Tollar et al., 2019), increase vitality (Labott & Donath, 2023), and improve brain health (Bray, Pieruccini‐Faria, Witt, Bartha, et al., 2023; Montero‐Odasso et al., 2023). Measuring agility throughout the lifespan is challenging; typical sport‐related agility field tests timed with stopwatches may be inappropriate for older adults (Coelho‐Junior et al., 2018; Miyamoto et al., 2008; Pauole et al., 2000; Young et al., 2021). Tests that have been used to measure agility in aging include but are not limited to, the Timed Up‐and‐Go (Barry et al., 2014; Coelho‐Junior et al., 2018; Podsiadlo & Richardson, 1991), the Ten Step (Miyamoto et al., 2008), and the Agility Challenge for the Elderly (Lichtenstein et al., 2019). Drawbacks of such tests are that they may lack significant challenge, especially for non‐clinical aging adults, and they do not consider movement quality, given that the primary outcome is completion time. Currently, there is no gold standard agility measure for aging adults.

Propulsive bipedal hopping, which involves an explosive, repetitive, horizontal jumping motion, measures agility given that it comprises the necessary components (i.e., strength, power, endurance, balance and coordination, and faster reaction time). Clinical agility tools incorporate hopping or hopping elements to detect impairment, such as the Berg Balance Scale (Blum & Korner‐Bitensky, 2008), Gross Motor Function Classification System (Palisano et al., 1997), and the Community Balance and Mobility Scale (Knorr et al., 2010); however, these tests technically target unique (clinical) populations, and each calculates hopping differently, making between‐test comparisons challenging. Exploring hopping spatiotemporal variables could provide further insight, potentially uncovering covert changes indicative of future impairment (Kirkland et al., 2017, 2018, 2022).

Several gait studies demonstrate that analyzing spatiotemporal values via an electronic instrumented walkway provides more insight than just speed or completion time, especially for those experiencing subtle or prodromal neuromuscular changes. For example, among older adults at risk of a dementia syndrome, stride time variability was positively associated with the risk of injurious falls (Pieruccini‐Faria et al., 2020) and dual‐tasking altered gait variability more than speed (Montero‐Odasso et al., 2020). Initial work from our group indicates that examining stride length, time, and variability uncover gait abnormalities among multiple sclerosis patients despite low disability scores (Chen et al., 2020). Further, propulsive bipedal hopping completed on an electronic walkway detected subtle lower limb (agility) impairments in older adults and individuals living with multiple sclerosis (Kirkland et al., 2017, 2018, 2022). More specifically, people with multiple sclerosis showing “normal” neurological exams and unimpaired walking demonstrated covert decline in agility performance, uncovered through hopping (Kirkland et al., 2017, 2018). No previous work has explored if propulsive bipedal hopping on an instrumented walkway is useful in detecting subtle age‐related agility deficits across the aging spectrum or the underlying neuromuscular changes that may be responsible for the deterioration of agility.

Transcranial Magnetic Stimulation (TMS), a non‐invasive brain stimulation technique, probes corticospinal tract (CST) function, the main motor pathway that controls trunk and limb movement (Chaves, Snow, et al., 2021). TMS generates electrical currents over the motor cortex to induce motor‐evoked potentials (MEPs) in targeted muscles, which are measured via electromyography (Rossini et al., 2015). Using TMS, several age‐related studies identified changes, such as altered CST excitability, lower MEPs, and reduced voluntary muscle activation (Hassanlouei et al., 2017; Rozand et al., 2019; Swanson & Fling, 2020). Similarly, age‐related decline at the muscular level is well documented. For example, altered muscle architecture via changes in fiber characteristics (Wu et al., 2016), and subsequent muscle atrophy or sarcopenia is a global health concern for all older adults (Keevil & Romero‐Ortuno, 2015). Whether CST function (i.e., upstream neurological system) or muscle strength (i.e., downstream muscle system) predicts age‐related differences in agility has not been examined; exploring such underlying physiology could enhance our understanding of the covert clinical manifestations detected by propulsive bipedal hopping on an instrumented walkway and, subsequently better target effective intervention strategies.

To this end, we recruited a healthy group of non‐athletic middle‐ to older‐aged adults to determine: (1) If aging is associated with propulsive bipedal hopping spatiotemporal properties and (2) If CST function or muscle strength more strongly predicts performance. We hypothesized that (1) aging negatively impacts these properties and (2) both muscle strength and CST function significantly contribute.

## 2 MATERIALS AND METHODS

### 2.1 Participants & sample size

After receiving Health Research Board approval (#2021.210), participants provided informed written consent to attend a ~2‐h visit, where we collected demographics, muscle strength, CST function, and propulsive bipedal hopping (more details below). Participants met the following inclusion criteria: (Morat et al., 2020) ≥ 30 years of age; (McNeil & Rice, 2018) able to jump forward two times consecutively; (Wu et al., 2016) no pain, inflammation, or sprains in any region of the lower body; (Hunter et al., 2016) able to walk indoors without an assistive gait device (e.g., cane, walker, etc.); (Swiecicka et al., 2019) no prior surgeries on their back or lower body; (Cadore et al., 2019) not a competitive athlete in a high‐performance sport (e.g., marathon runner, triathlon athlete, etc.); (Buch et al., 2016) met all TMS safety requirements (Rossini et al., 2015); and (Lichtenstein et al., 2020) walking speed ≥120 cm/s, a safe minimum speed for community ambulation (Salbach et al., 2014).

We calculated the sample size using G*Power software, Version 3.1.9.4 (Aichach, Germany). According to a regression analysis with alpha set at 0.05, power at 0.80, a large effect size (f2 = 0.35), and three predictors, we required a minimum of 20 participants. However, we exceeded this value (*n* = 32) to allow for incomplete data and outliers.

### 2.2 Assessment

#### 2.2.1 Demographics

We collected participants' chronological age (years) and biological sex (i.e., sex assigned at birth) via standard questionnaires. We measured height (centimeters; cm), weight (kilograms; kg), and body mass index (kg/m^2^) using a Health‐O‐Meter Professional scale (McCook, IL, USA). Using a tape measure, we calculated leg length (cm) from the anterior superior iliac spine to the medial malleolus (Bolz & Davies, 1984).

#### 2.2.2 Quadriceps strength

We quantified muscle strength via the quadriceps, given its role in hopping as the primary knee extensor (National Strength and Conditioning Association, 2021). We measured quadriceps strength using the Manual Muscle Tester (Unit 01165, Lafayette Instrument, Lafayette, IN, USA). Participants sat on a height‐adjustable plinth table (SEERS Medical Ltd., United Kingdom) with their arms placed across their chest and legs hanging over the edge. The examiner positioned the muscle tester across the anterior tibia, ~5 cm superior to the malleolus, using a strap looped through the dynamometer and secured to the table leg. As such, the participant's knee was flexed to ~90°. Participants performed a maximum voluntary contraction after being instructed to “extend at the knee as hard and as fast as possible for five seconds.” We provided verbal encouragement to each participant for the duration of the contraction. Force outputs during contractions were measured in kg. The testing order was randomized, and the participant completed the protocol twice on each leg, with all four bilateral scores averaged into a single‐leg strength score. Previous work has demonstrated that this leg strength protocol possesses strong validity (Lesnak et al., 2019).

#### 2.2.3 Measuring corticospinal tract function using transcranial magnetic stimulation

Our TMS protocol required pinch strength (kg), assessed using a pinch gauge (B&L Engineering, Santa Ana, CA, USA), to determine the participant's weaker hand. The full TMS protocol is available in our previous work (Chaves, Snow, et al., 2021), but, in brief, we started by cleaning and debriding the skin surface before attaching surface electromyography electrodes (Kendall 200 Covidien, Mansfield, MA) over the first dorsal interosseus muscle (active electrode), the proximal interphalangeal joint of the index finger (reference electrode), and the ulnar styloid process (ground electrode). The first dorsal interosseus muscle is prominently used to measure active motor threshold in healthy individuals (Houde et al., 2018; Rozand et al., 2019), and previous works indicate that TMS variables derived from the upper limb represent overall CST function (Chaves et al., 2024; Chaves, Kenny, et al., 2021; Singh et al., 2014; Walsh et al., 2019). A built‐in electromyography system used 2500 V/V amplification collected at a 3 kHz sampling rate with a gain of 600 V/V and a bandwidth of 16–550 Hz. Electromyography data were assessed using Signal Software, version 6.06 (Cambridge Electronic Design Ltd., Cambridge, UK).

Per previous recommendations, we targeted the hemisphere corresponding to the weaker hand (i.e., lower pinch strength) (Chaves, Snow, et al., 2021). With the participant in a seated position, we used Neuronavigation (Brainsight, Rogue Research Inc., Montreal, QC, Canada) to guide head and coil positioning. Using monophasic magnetic posterior–anterior pulses from a BiStim 2002 stimulator (Magstim Co. Whitland, UK), we administered single‐pulse TMS with the coil placed tangentially to the scalp and held at a 45° angle from the midline perpendicular to the central sulcus. We stimulated positions along the primary motor area at a supra‐threshold intensity to determine the motor “hotspot” or the location with the greatest response per MEP peak‐to‐peak amplitude measured in microvolts (μV) (Chaves, Snow, et al., 2021).

After identifying the hotspot, we performed two TMS protocols. First, the active motor threshold protocol required the participant to hold 10% of their maximum voluntary contraction pinch, as determined via the pinch gauge test. The active motor threshold represents the maximum stimulator output obtained in five out of 10 MEP responses ≥200 μV. A higher active motor threshold indicates lower excitability and, therefore, reduced CST function. Second, we executed a recruitment curve protocol by stimulating participants six times each at six supra‐threshold intensities (36 separate stimulations): 105%, 115%, 125%, 135%, 145%, and 155% of the active motor threshold. For each pulse, 1‐s‐long sweeps collected electromyography activity from 100 ms before the TMS pulse to 900 ms afterwards. We calculated the excitatory recruitment curve using the average MEP amplitudes during the six supra‐threshold intensities (Chaves et al., 2019; Chaves, Snow, et al., 2021). Similarly, we calculated the inhibitory recruitment curve by taking the average cortical silent period (CSP) at each supra‐threshold intensity (Chaves et al., 2019; Chaves, Snow, et al., 2021). The cortical silent period represents the time (ms) between MEP onset (time of MEP exceeding ±2SD from background electromyography activity) and returning electromyography activity (Rossini et al., 2015). Using the trapezoid integration technique, we determined the area under the excitatory and inhibitory MEP recruitment curves (Potter‐Baker et al., 2016; Talelli et al., 2008). The excitatory MEP recruitment curve reflects voltage‐gated ion channel and glutamatergic activity (Rossini et al., 2015; Ziemann et al., 2015), while the inhibitory MEP recruitment curve indexes the excitability of GABAergic corticospinal neurons (Rossini et al., 2015; Ziemann et al., 2015). In summary, our TMS variables of interest were the (1) (excitatory) active motor threshold, (2) area under the curve for the excitatory recruitment curve, and (3) area under the curve for the inhibitory recruitment curve.

#### 2.2.4 Propulsive bipedal hopping agility test

Before proceeding to the propulsive bipedal hopping agility test, we measured participants “comfortable” gait speed (cm/s) via a ~15 feet (length) by ~3 feet (width) ProtoKinetics Zeno Walkway (ProtoKinetics LLC, Havertown, PA) and ProtoKinetics Movement Analysis Software (PKMAS; ProtoKinetics LLC, Havertown, PA). Before executing a gait trial, participants received a demonstration from the assessor. Participants completed two lengths (i.e., up and back), starting, finishing, and turning ~3 feet before/after the walkway end (i.e., the marked X) to avoid recording acceleration/deceleration phases. Two walking trials were completed with approximately 1 min of rest in between trials (Chen et al., 2020). Incomplete walkway footfalls were removed for calculations of comfortable gait speed. Participants with a gait speed <120 cm/s did not proceed to the agility test.

**FIGURE 1:**
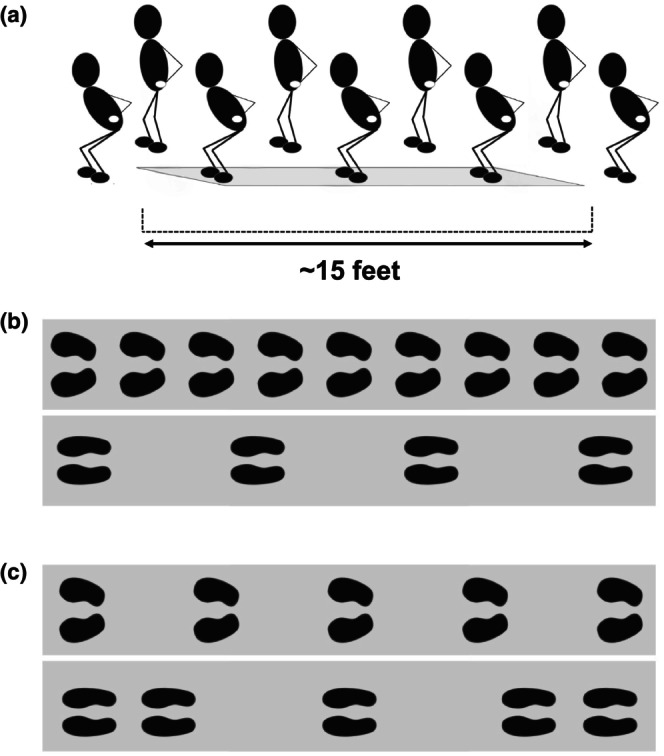
Panel (a): Graphical representation of the bipedal propulsive hopping protocol. Participants began and finished the task just before/after the start/finish of the mat. Panel (b): Example of participants with different hop lengths (top is a shorter length than bottom). Panel (c): Example of participants with different hop length variability (top is less variable or more consistent than bottom).

Using the same walkway, participants performed a propulsive bipedal hopping agility test via multiple forward displacement jumps with both feet synchronously (i.e., leaving and returning to the ground together) (Kirkland et al., 2022). Following a demonstration by the examiner, we instructed participants to place their hands on their hips and “hop as fast and safely as possible.” Due to the physical demands, participants performed only one trial. However, participants could restart if they made an error, such as landing on the sides of the walkway where only partial footfalls are recorded or failing to complete the proper hopping technique. Hopping variables extracted from the walkway included: (1) hop length (cm) or the distance from the heel contact of one hop to the heel contact of the following hop; and (2) hop length variability as per the coefficient of variation for hop length or the difference of all hop lengths (Figure 1). To account for anthropometric differences, we adjusted hop length for leg length (Davies et al., 1988; Kirkland et al., 2022). We visually recorded all hopping tests with a Logitech C920 Carl Zeiss HD 1080p Webcam (Logitech, San Jose, CA) to qualitatively confirm hopping accuracy.

### 2.3 Statistical analysis

We conducted statistical analyses in IBM SPSS Statistics, version 29 (IBM Corp, Armonk, NY), with a *p*‐value <0.05 indicating statistical significance. With the exception of sex, we report participant characteristics (i.e., age, height, weight, body mass index, and leg length) and outcomes (i.e., gait speed, quadriceps strength, hopping variables, and CST function) as mean ± standard deviation.

First, we identified outliers in our variables of interest as (raw) data points ± 3 times the interquartile range—no participants were flagged. We used Pearson correlations to clarify relationships between age, quadriceps strength, CST function (i.e., active motor threshold and area under the curve for excitatory and inhibitory recruitment curves), and bipedal hopping variables (i.e., hop length and hop length variability). Correlations between variables were considered weak at *r* < 0.30, moderate at 0.30 < *r* < 0.70, and strong at *r* > 0.70. Only independent variables (i.e., quadriceps strength and CST function) demonstrating significant correlations with dependent variables (i.e., hop length and hop length variability) proceeded to the regression analyses.

Model 1 of our regression controlled for a single covariate, sex; we did not control for age as we expected it to impact the dependent variables given the age range of included participants. Model 2 included sex and a single independent variable; we repeated this approach for every independent variable that demonstrated a significant correlation with our dependent variables. We ensured all regressions satisfied the following assumptions prior to execution: (1) Independence of observations via a Durbin‐Watson statistic ranging from 1.5 to 2.5 (Draper & Smith, 1998); (2) Collective linearity of the independent variables or predictors, as well as homoscedasticity, via visual inspection of a scatterplot containing studentized residuals and (unstandardized) predictive values; (3) Linearity of each independent variable or predictor via visual inspection of partial regression plots; (4) Absence of multicollinearity via a tolerance value >0.1 and VIF value <10 (Hair et al., 2014); 5) No unusual points, including outliers via studentized deleted residuals values > ± 3 and leverage points >0.2 or influential points via cook's distance >1 (Cook & Weisberg, 1982); and (6) Normality via visual inspection of the standardized residuals histogram and Q‐Q plot.

## 3 RESULTS

### 3.1 Demographics & correlations

**FIGURE 2:**
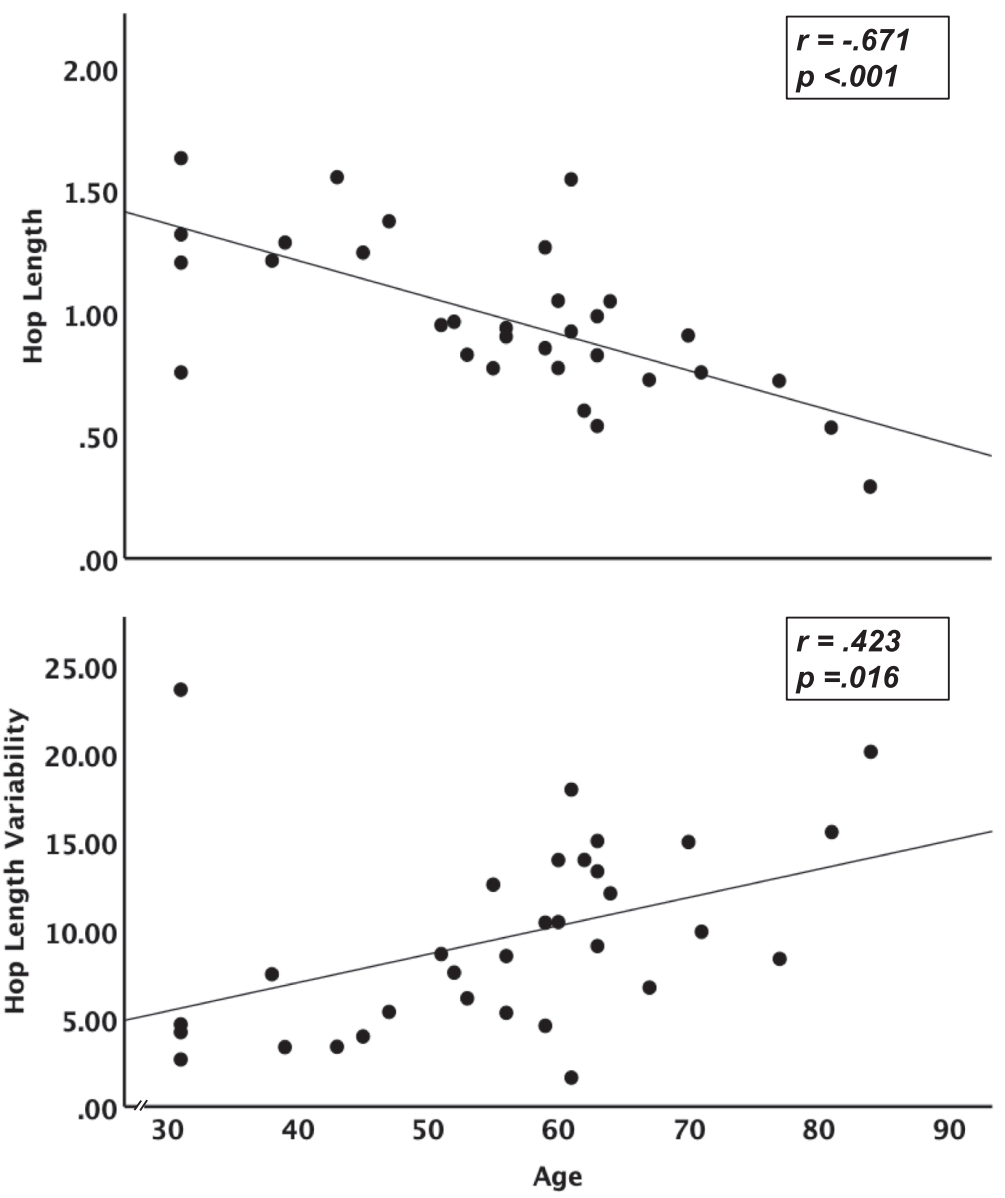
Age (measured in years) is associated with shorter leg‐length‐adjusted hop length (top panel) and a worsening in hop length variability (measured as a coefficient of variation; bottom panel).

**FIGURE 3:**
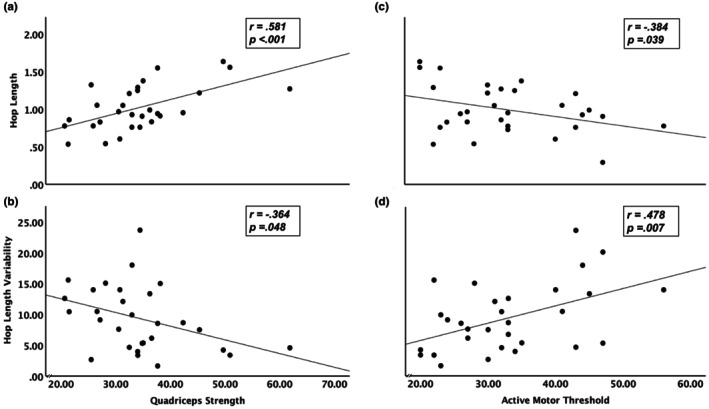
Stronger quadriceps (measured in kilograms) were significantly correlated with longer leg length‐adjusted hop length (Panel a) and lower or better hop length variability (measured as coefficient of variation; Panel b). Similarly, lower or better active motor threshold (measured as the percentage of maximum stimulator output or %MSO) was significantly correlated with longer leg length‐adjusted hop length (Panel c) and lower or better hop length variability (Panel d).

Our 32 participants (14 male, 18 female) were, on average, 55.75 ± 14.22 years of age, with the youngest and oldest being 31 and 84, respectively (Table 1). Age was moderately correlated with hop length (*r* = −0.671, *p* < 0.001) and hop length variability (*r* = 0.423, *p* = 0.016) (Figure 2). Hop length and hop length variability were moderately correlated with quadriceps strength (Figure 3 Panel a: *r* = 0.581, *p* < 0.001; Figure 3 Panel b: *r* = −0.384, *p* = 0.039) and active motor threshold (Figure 3 Panel c: *r* = −0.364, *p* = 0.048; Figure 3 Panel d: *r* = 0.478, *p* = 0.007). We found no significant correlations between the excitatory or inhibitory recruitment curves and hop length or hop length variability (*p* > 0.644). Therefore, we did not progress excitatory and inhibitory recruitment to the regression analysis.

**TABLE 1:** Participant characteristics.

Characteristic	Mean ± SD	Range
*n* (female)	32 (18)	
Age (Years)	55.75 ± 14.22	31–84
Height (centimeters)	168.35 ± 9.12	155.50–187.60
Weight (kilograms)	74.80 ± 18.39	49.10–138.40
Body Mass Index	26.12 ± 4.67	19.97–41.78
Leg Length (centimeters)	87.33 ± 6.71	74.00–102.00
Gait Speed (centimeters/second)	171.62 ± 20.78	132.05–216.12
Quadriceps Strength (kilograms)	34.37 ± 9.18^a^	20.68–61.85
Hop Length (centimeters)	85.51 ± 30.68	24.73–155.04
Hop Length Variability (CV)	9.56 ± 5.42	1.64–23.65
Active Motor Threshold (%MSO)	33.03 ± 9.35^b^	20–56
Excitatory REC AUC	51,724.48 ± 16,959.65^b^	24,995‐87,970
Inhibitory REC AUC	5243.23 ± 1229.38^b^	2896‐7447

Abbreviations: % MSO, percentage of maximum stimulator output; AUC, area under the curve; Body mass index, weight (kilograms)/height^2^ (centimeters); CV, coefficient of variation; REC, recruitment curve; SD, standard deviation.

^a^ *n* = 29.

^b^ *n* = 30.

### 3.2 Quadriceps strength predicts hop length, while active motor threshold predicts hop variability

After controlling for biological sex, we found quadriceps strength significantly explained 31.1% of leg length‐adjusted hop length (*R* ^2^ = 0.345, *p* = 0.002; Table 2). Therefore, for every one‐kilogram increase in quadriceps strength, hop length increased by 0.408 cm. Active motor threshold did not significantly explain hop length (*R* ^2^ = 0.143, *p* = 0.075; Table 2).

**TABLE 2:** Quadriceps Strength but not Active Motor Threshold predicts leg‐length adjusted hop length.

Model	Variables	*B*	*R* ^2^	Adjusted *R* ^2^	*R* ^2^ change	F change	Sig. F change
1	Sex	0.866	0.034	−0.002	0.034	0.953	0.338
2	Sex and Quadriceps Strength^a^	0.408	0.345	0.294	0.311	12.331	0.002
1	Sex	0.821	0.034	−0.001	0.034	0.980	0.331
2	Sex and Active Motor Threshold^b^	1.281	0.143	0.080	0.109	3.441	0.075

*Note*: Dependent Variable: Leg length‐adjusted hop length (measured in centimeters). Independent Variables: Sex, Quadriceps Strength (measured in kilograms), and Active Motor Threshold (measured in percentage of maximum stimulator output or %MSO). *Β* = Unstandardized beta.

^a^ *n* = 29.

^b^ *n* = 30.

After controlling for sex, we found quadriceps strength did not significantly explain hop variability (*R* ^2^ = 0.148, *p* = 0.069; Table 3). However, the active motor threshold, measured in percentage of maximum stimulator output, significantly explained 19.6% of hop length variability (*R* ^2^ = 0.239, *p* = 0.014; Table 3). Therefore, for every one‐unit increase in active motor threshold, hop length variability increased by 2.157.

**TABLE 3:** Active Motor Threshold but not quadriceps strength predicts leg length‐adjusted hop length variability.

Model	Variables	*B*	*R* ^2^	Adjusted *R* ^2^	*R* ^2^ change	F change	Sig. F change
1	Sex	11.924	0.030	−0.006	0.030	0.832	0.370
2	Sex and Quadriceps Strength^a^	16.974	0.148	0.082	0.118	3.597	0.069
1	Sex	12.643	0.042	0.008	0.042	1.239	0.275
2	Sex and Active Motor Threshold^b^	2.157	0.239	0.182	0.196	6.956	0.014

*Note*: Dependent Variable: Hop length variability (measured as coefficient of variation). Independent Variables: Sex, Quadriceps Strength (measured in kilograms), and Active Motor Threshold (measured in percentage of maximum stimulator output or %MSO). *Β* = Unstandardized beta.

^a^ *n* = 29.

^b^ *n* = 30.

## 4 DISCUSSION

We undertook this study to determine whether performance on a propulsive bipedal hopping agility task was associated with aging and was predicted by muscle strength and/or CST function. We report three main findings. In support of our hypothesis, older age is indeed associated with shorter hop length and greater (i.e., worse) hop length variability, supporting the use of propulsive bipedal hopping to detect age‐related changes in agility. In partial support of our hypothesis, greater quadriceps strength predicted longer hop length, but higher CST excitability did not. Also in partial support of our hypothesis, lower (i.e., worse) CST excitability (i.e., higher active motor threshold) predicted greater (i.e., worse) hop variability, but quadricep strength did not. Therefore, stronger quadriceps increased the distance hopped, and higher CST excitability led to less variability or more consistency in hop length.

Our propulsive bipedal hopping task offers a novel, dynamic, and intricate assessment of agility by integrating key agility components (i.e., strength, power, endurance, balance and coordination, and reaction time) into a simple and easily administered test. Further, executing the propulsive bipedal hopping task on an electronic instrumented walkway permits the extraction of spatiotemporal properties that offer unique insight into subtle but meaningful performance variations. As an agility outcome, we theorize that our propulsive bipedal hopping task may be best suited for exposing covert but meaningful differences in non‐clinical older adults, those in transitional stages (i.e., mild cognitive impairment), and even mid‐life adults; in recent years, it has become increasingly apparent that health or lack thereof in mid‐life significantly influences outcomes in older age (Deng et al., 2024; Kivipelto et al., 2001; Rovio et al., 2005). In contrast, conventional agility tests, such as the Timed Up‐and‐Go (Barry et al., 2014; Coelho‐Junior et al., 2018; Podsiadlo & Richardson, 1991) and the Ten Step test (Miyamoto et al., 2008), may remain better suited for the oldest old (75+ years of age) (Wallace et al., 2021) and/or clinical populations due to their increased fall risk. Future work should compare the utility of our propulsive bipedal hopping task to other agility tests across the aging spectrum and/or in clinical context when safe to do so.

Across the aging spectrum, we believe we have identified components that individually represent two aspects of movement: (1) hop length, which is driven by the muscle system's ability to propel the body; and (2) hop variability, which involves control and coordination, representing the central nervous system's integration of sensory and proprioceptive inputs into smooth, fluid actions. Previous research suggests that higher or better CST function strongly correlates with greater lower extremity strength in stroke patients (Madhavan et al., 2011). However, more recent studies using methodologies more closely aligned with our own indicate that the relationship between CST function and lower‐extremity strength is attenuated when controlling for age, albeit in people living with multiple sclerosis (Baird et al., 2018). Together, our findings and Baird and colleagues (Baird et al., 2018) emphasize the overlapping but distinct roles of neurological (i.e., CST function) and muscular components in the neuromuscular system. At the very least, our findings suggest that muscle weakness or atrophy, better known as sarcopenia (Jentoft et al., 2019), significantly affects hop length and, by extension, agility. Importantly, sarcopenia provides a direct avenue for intervention. Physical exercise, specifically resistance training (i.e., weightlifting) and nutrition/supplementation are widely regarded as the two most effective approaches to combat muscle/strength loss (Cruz‐Jentoft et al., 2014; Yoshimura et al., 2017). Importantly, the prescription differs slightly when considering the neurological contributions.

The ascending and descending neural pathways relay sensory or motor info to and from the cerebral cortex, thereby enhancing proprioception to promote efficient motor movements (Porter & Lemon, 1995). Due to the pathways' complexity, which includes lower motor neurons, interneurons, and the cerebral cortex, there are multiple opportunities for signal transmission and, thereby, CST function to be altered or degraded (Lemon, 2008; Welniarz et al., 2017). Discerning the contributions of each component to signal degradation is difficult but possible (Taylor, 2006). However, it is made more challenging because the exact brain regions responsible for specific movements are not yet fully understood. For example, the primary motor cortex is central to initiating movement (Canedo, 1997; Kakei et al., 1999), but the cerebellum plays an essential role in ensuring that movements are smooth, coordinated, and precise in tasks that require millimeter accuracy (Manto et al., 2012). Our findings underscore that the neurological component of the neuromuscular system, and not muscle (strength), is pivotal in hop variability. Like the link between muscle strength and hop length, such a result provides an opportunity for targeted intervention prescription. The research is not as definitive as sarcopenia prevention, but again, resistance training demonstrates efficacy in improving CST function (Gomez‐Feria et al., 2023; Kidgell et al., 2017). However, CST function may also benefit from targeted skills training for coordination and reaction time; such training is possible in the real world, but it has become increasingly popular via “games” in robotic or virtual reality devices, especially in clinical populations (Pourazar et al., 2018; Zhu et al., 2021). Other intervention strategies to improve CST function include repetitive TMS (Dall'Agnol et al., 2014), neurofeedback (Liang et al., 2020), vitamin D supplementation (Al‐Amin et al., 2019), and more.

### 4.1 Study limitations & future directions

In addition to the limitations and forward directions already put forth, we would be remiss not to highlight that our strength measure focused on knee extension, which is primarily executed by the quadriceps muscles (National Strength and Conditioning Association, 2021). However, hopping is a full‐body movement, with motion primarily generated by the lower body muscles. Therefore, other muscles and the movements they generate also play a critical role in hopping, such as the hip extensors, knee flexors, and ankle plantar flexors. Exploring the muscle strength of these movements relative to hop length and hop length variability may provide complementary insight; this is especially true for the gluteus maximus, given that it is the largest muscle in the human body (Ito et al., 2003). Additionally, the core plays a critical role in transferring force to the extremities (Notte et al., 2024), and there is evidence that it can improve jump performance (Lee et al., 2024; Ozmen, 2016); in our hopping task, participants were required to keep their hands on their hips, which likely impaired balance, making core stability an important contributor to performance. Measuring muscle power instead of muscle strength may also provide greater insight. Strength and power are similar, but the former measures absolutes, whereas the latter includes a time component. Seminal research (Reid & Fielding, 2012), as well as complementary work (Katula et al., 2008; Simpkins & Yang, 2022), suggests that muscle power is a more sensitive and clinically meaningful measure than strength in old age. Along these lines, applying machine learning to the electronic walkway data may reveal novel and insightful variables not extracted using conventional approaches (Hu et al., 2022a, 2022b). Another limitation is that our regression only considered three independent variables: sex, quadriceps strength, and CST function, with the latter two selected because of our interest in the individual components of the neuromuscular system. It is plausible that other factors, especially those beyond the neuromuscular system, play a role in hop length and hop length variability during a propulsive bipedal hopping task. In this regard, our analysis simply controlled for sex, which is different than stratifying for sex or performing a sex‐specific analysis. It is increasingly evident that the aging experience is sex‐specific, with differences observed at the molecular (Titus et al., 2021), cellular (Bray, Pieruccini‐Faria, Witt, Rockwood, et al., 2023), and clinical levels (Gordon et al., 2017). Future work should be adequately powered to explore the sex‐aging interaction in the neuromuscular contributions to agility.

## 5 CONCLUSIONS

In the current study, we examined the relationships between performance in a propulsive bipedal hopping agility task and the neuromuscular system in a sample of healthy non‐athletes who were over 30 years of age and had no walking impairments. Our results indicate a strong correlation between age and our hopping spatiotemporal variables (i.e., leg‐length‐adjusted hop length and hop length variability). However, after controlling for sex, muscle strength predicted hop length, while CST function predicted hop length variability. This study provides evidence that propulsive bipedal hopping is a robust indicator of age‐related adaptations, capable of revealing subtle differences in neuromuscular function.

## AUTHOR CONTRIBUTIONS

EGM: Conceived and designed research, performed experiments, analyzed data, interpreted results of experiments, prepared figures, edited and revised manuscript, approved final version of manuscript. NWB: Conceived and designed research, analyzed data, interpreted results of experiments, prepared figures, drafted manuscript, edited and revised manuscript, approved final version of manuscript. SZR: Performed experiments, analyzed data, approved final version of manuscript. CJN: Performed experiments, approved final version of manuscript. HMM: Performed experiments, approved final version of manuscript. MP: Conceived and designed research, analyzed data, interpreted results of experiments, edited and revised manuscript, and approved final version of manuscript.

## FUNDING INFORMATION

Nick W. Bray, Faculty of Medicine, The Canadian Institutes of Health Research Fellowship: FRN – 489,847. Michelle Ploughman, Faculty of Medicine, The Canadian Institutes for Health Research: 169649 and 173,526; Newfoundland and Labrador Research and Development Corporation: 5404.1699.104; Canada Foundation for Innovation: 33621.

## CONFLICT OF INTEREST STATEMENT

The authors declare no conflicts of interest. The sponsors had no role in the study design, collection, analysis and interpretation of data, writing of the report or decision to submit the article for publication.

## ETHICS STATEMENT

The institutional ethics board approved the study (Health Research Board approval #2021.210), and the study complied with the Declaration of Helsinki.

## Data Availability

Data available upon reasonable request to the corresponding author (Michelle Ploughman, PT, PhD – michelle.ploughman@med.mun.ca) or the co‐first author (Nick W. Bray, PhD – nwbray@mun.ca).
